# Assessment of Risk Factors for Vasoplegic Syndrome in Patients Undergoing Cardiac Surgery With Cardiopulmonary Bypass

**DOI:** 10.7759/cureus.90758

**Published:** 2025-08-22

**Authors:** Kristin A Meyer, Heidi L Brink, Stephen C Brannan, Scott W Lundgren, Elizabeth R Lyden, Quinton B Behlers

**Affiliations:** 1 Pharmacy, Nebraska Medicine, Omaha, USA; 2 Anesthesiology and Critical Care, University of Nebraska Medical Center, Omaha, USA; 3 Cardiovascular Medicine, University of Nebraska Medical Center, Omaha, USA; 4 Biostatistics, University of Nebraska Medical Center, Omaha, USA

**Keywords:** angiotensin ii, cardiac surgery, cardiopulmonary bypass, hydroxocobalamin, methylene blue, risk factors, vasoplegic syndrome

## Abstract

Introduction: Vasoplegic syndrome (VS), a common complication of patients undergoing cardiac surgery with cardiopulmonary bypass (CPB), can be defined as an abnormally low systemic vascular resistance presenting as profound hypotension and vasodilatory shock despite adequate cardiac output. Multiple risk factors for the development of VS post-CPB have been explored. The purpose of this study is to identify additional patient-specific risk factors associated with VS, including angiotensin-converting enzyme (ACE) inhibitors, angiotensin II receptor blockers, angiotensin receptor/neprilysin inhibitor, beta blockers, dihydropyridine calcium channel blockers, dobutamine, milrinone, HMG-CoA reductase inhibitors, aspirin, P2Y12 inhibitors, scheduled phosphodiesterase 5 inhibitors, intravenous treprostinil, hydralazine, sulfonylureas, continuous infusion loop diuretics, vasopressors, other anti-hypertensive agents, amiodarone, and steroid use.

Methods: This single-center, retrospective, case-control study includes adult patients ≥19 years old admitted to the cardiovascular intensive care unit (ICU) following cardiothoracic surgery with CPB. Patients in the VS group were identified as having received methylene blue, hydroxocobalamin, or angiotensin II within 48 hours of CPB initiation. Patients were excluded if they were pregnant, incarcerated, or <19 years old. Controls were matched by procedure type, age within five years, and patient sex. The primary outcome is a correlation of risk factors of VS. Secondary outcomes include vasopressor utilization at pre-specified time points, ICU length of stay, hospital length of stay, and survival to hospital discharge. Statistical analyses used Fisher’s exact test for categorical data and the Wilcoxon rank-sum test or independent samples t-test for continuous data (PC SAS v9.4, significance at p<0.05).

Results: Of 63 patients identified as having received methylene blue, hydroxocobalamin, or angiotensin II, 38 patients met the inclusion criteria. After matching, 114 patients were included in the analysis. Baseline characteristics were similar, with 83% male patients and a median age of 57.5 years, except for a difference in CPB duration (189 minutes vs. 142.5 minutes, p=0.017). When assessing risk factors for the primary outcome, there was no difference between groups except with hydralazine (9 (23.7%) vs. 7 (9.2%), p=0.047). There were differences observed in ICU length of stay (7 vs. 4 days, p=0.004), hospital length of stay (25 vs. 22.5 days, p=0.028), and survival to hospital discharge (28 (73.7%) vs. 75 (98.7%), p<0.001).

Conclusions: Hydralazine was the only pre-operative factor identified with a higher incidence of usage in the VS group. Further research is needed to determine the clinical significance of this association.

## Introduction

Vasoplegic syndrome (VS) is defined as an abnormally low systemic vascular resistance (<800 dyne s/cm^5^) presenting as profound hypotension (mean arterial pressure <65 mmHg) and vasodilatory shock despite adequate cardiac output (cardiac index >2.2 L/min/m^2^) [1]. Fluid resuscitation and administration of vasopressors are often required to maintain end-organ perfusion [1]. VS is recognized as a common complication of patients undergoing cardiac surgery, particularly with cardiopulmonary bypass (CPB), with an incidence ranging from 5% to 25% [2]. VS is associated with high morbidity and mortality, with mortality rates as high as 25% being reported [3].

The mechanism underlying post-CPB VS is multifactorial, but is believed to be associated with an immunologic response characterized by the release of pro-inflammatory mediators, complement activation, ischemia-reperfusion injury, blood transfusion, and/or exposure of the blood to the foreign surfaces of the CPB [4]. This immunologic response leads to elevated levels of oxygen-free radicals, endothelins, nitric oxide (NO), platelet-activating factors, thromboxane A2, prostaglandins, and various cytokines [4].

Multiple agents have been utilized in the setting of VS, including, but not limited to, methylene blue, hydroxocobalamin, and angiotensin II [1,2,5,6]. Methylene blue works by competing with NO and blocking cyclic guanosine monophosphate (cGMP) accumulation, leading to restoration of vascular tone [2]. Hydroxocobalamin inhibits inducible NO synthase and scavenges NO directly, reducing vasodilation and elevating blood pressure [5,6]. Angiotensin II is a naturally occurring hormone in the renin-angiotensin-aldosterone system (RAAS) that acts as a potent vasoconstrictor of both arteries and veins to increase blood pressure [7,8]. Administration of modified bovine angiotensin II has been shown to elicit consistent vasopressor effects in patients with vasodilatory shock [8].

Multiple risk factors for the development of VS post-CPB have been explored [3,9]. In particular, existing studies attempted to determine an association between specific medications and conflicting evidence. Medications previously evaluated include angiotensin-converting enzyme (ACE) inhibitors, angiotensin receptor blockers (ARB), beta blockers, inotropic medications, and more [3,9]. This study seeks to validate existing risk factors and identify additional risk factors of VS in patients undergoing cardiac surgery with CPB.

## Materials and methods

This single-center, retrospective, case-control study includes adult patients ≥19 years old admitted to the cardiovascular ICU following cardiothoracic surgery with CPB from June 2020 to September 2024. Patients were excluded if they were pregnant, incarcerated, or <19 years old. Patients in the VS group included any patient who received methylene blue, hydroxocobalamin, or angiotensin II within 48 hours of CPB initiation. The control group included those who underwent the same procedures without the receipt of these agents. Subjects were matched in a 1:2 ratio case:control by procedure type, age within five years, and sex. The study was approved by the University of Nebraska Medical Center Institutional Review Board (approval number: 0603-24-EP) with consent waived.

The primary outcome was a correlation of risk factors of VS. Risk factors assessed included receipt of the following agents within the 72 hours prior to CPB initiation: ACE inhibitors, angiotensin II receptor blockers, angiotensin receptor/neprilysin inhibitor, beta blockers, dihydropyridine calcium channel blockers, dobutamine, milrinone, HMG-CoA reductase inhibitors, aspirin, P2Y12 inhibitors, scheduled phosphodiesterase 5 inhibitors, intravenous treprostinil, hydralazine, sulfonylureas, continuous infusion loop diuretics, vasopressors, and other anti-hypertensive agents. Other risk factors assessed included receipt of amiodarone within the past 30 days, receipt of a systemic steroid within the past year, and a left ventricular ejection fraction ≤40% or a durable left ventricular assist device (LVAD) in place before surgery. Secondary outcomes included vasopressor utilization at pre-specified time points, ICU length of stay, hospital length of stay, and survival to hospital discharge. Vasopressor utilization was calculated in norepinephrine equivalents (NEE) using the conversions from the ATHOS 3 trial [7]. Pre-specified time points included the highest dose utilized intra-operatively, ICU arrival, and 12-, 24-, and 48-hours post-CPB initiation.

Descriptive statistics (medians, IQR counts, and percentages) were used to summarize the data. Continuous data were compared between the groups using the independent samples t-test (if data met assumptions of normality) or the Wilcoxon rank-sum test (if data were considered non-normal). Fisher’s exact test was used to look at the association of categorical variables with a group. All analyses were done using SAS, Version 9.4 (SAS Institute, Cary, USA). A p-value <0.05 was considered statistically significant.

## Results

**Figure 1:**
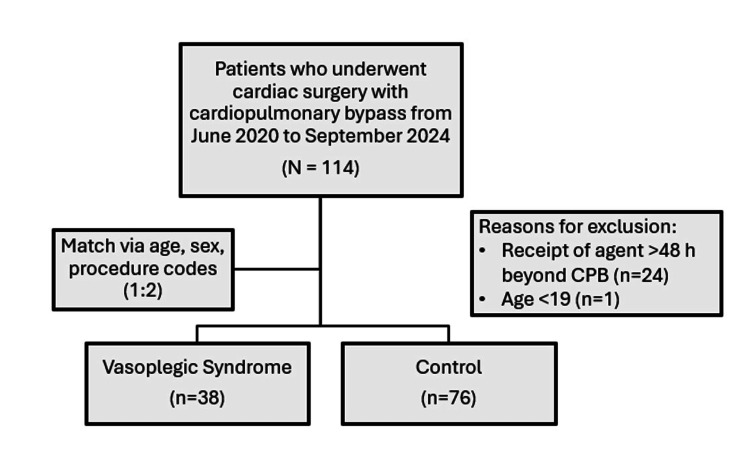
Study design

A total of 114 patients who underwent cardiac surgery from June 2020 to September 2024 were included. Sixty-three patients were identified by receipt of methylene blue, hydroxocobalamin, or angiotensin II. Twenty-four patients were excluded due to receipt of treatment >48 hours after CPB initiation, and one patient was <19 years old. This left 38 patients in the VS group, who were then matched to 76 patients in the control group (see Figure 1).

In the VS group, five (13%) patients underwent coronary artery bypass graft (CABG), two (5%) CABG with a valve replacement or repair, four (11%) valve replacement or repair only, 14 (37%) durable LVAD implantation, and 13 (34%) heart transplantations. The control group was matched by procedure type, with the exception of the two CABG patients with valve replacement or repair patients, as these were matched to CABG-only procedures. The median duration of CPB was longer in the VS group (189 minutes vs. 142.5 minutes, p=0.017). Additionally, those in the VS group had a higher weight compared to the control group (105 kg and 93 kg, respectively, p=0.044) and lower hemoglobin (10.4 vs. 12.0 g/dL, p=0.002), hematocrit (32.5 vs. 37.1%, p=0.011), and eGFR (59 vs. 65 mL/min/1.73 m^2^, p=0.013). There were no differences in the remaining baseline characteristics, which can be seen in Table 1.

**Table 1:** Baseline demographics *VS, n=24, and control, n=45, because left ventricular ejection fraction was reported only for patients with a diagnosis of heart failure without a durable LVAD +VS, n=14, and control, n=26, because this was the number of patients who required temporary MCS ^VS, n=8, and control, n=9, because this was the number of patients who required durable MCS ¹Fisher exact p-value; ²Wilcoxon rank-sum p-value; ³Unequal variance two-sample t-test CPB, cardiopulmonary bypass; ECMO, extracorporeal membrane oxygenation; IABP, intra-aortic balloon pump; LVAD, left ventricular assist device; MCS, mechanical circulatory support; VS, vasoplegic syndrome.

Variable	VS (n=38)	Control (n=76)	P-value
Sex, male - no. (%)	32 (84.2)	63 (82.3)	1.000^1^
Age, years - median (IQR)	57.5 (48-70)	57.5 (47.5-68.5)	0.668^2^
Weight, kg - median (IQR)	105 (84-119)	93 (80-113)	0.044^2^
History of liver disease - no. (%)	9 (23.7)	13 (17.1)	0.454^1^
History of heart failure - no. (%)	32 (84.2)	55 (72.4)	0.242^1^
Left ventricular ejection fraction, %* - median (IQR)	15 (10-20)	15 (10-20)	0.273^2^
History of cardiac surgery - no. (%)	17 (44.7)	24 (31.6)	0.215^1^
Hemoglobin, g/dL - median (IQR)	10.4 (8.6-12.0)	12.0 (10.5-14.1)	0.002^2^
Hematocrit, % - median (IQR)	32.5 (27.2-38.6)	37.1 (31.7-42.1)	0.011^2^
Serum creatinine, mg/dL - median (IQR)	1.34 (0.93-1.78)	1.12 (0.96-1.40)	0.050^2^
eGFR, mL/min/1.73 m^2^ - median (IQR)	59 (43-60)	65 (58-78)	0.013^2^
Pre-operative temporary MCS: IABP - no. (%)	12 (31.6)	18 (23.7)	0.376^1^
Pre-operative temporary MCS: Impella - no. (%)	5 (13.2)	11 (14.5)	1.000^1^
Pre-operative temporary MCS: ECMO - no. (%)	2 (5.3)	3 (3.9)	1.000^1^
Pre-operative durable MCS: LVAD - no. (%)	8 (21.1)	9 (11.8)	0.264^1^
Time on pre-operative temporary MCS, days^+ ^- median (IQR)	17 (9.5-22)	9 (2-18)	0.071^2^
Time on pre-operative durable MCS (LVAD), days^ - median (IQR)	1342 (737-1528)	667 (532-1649)	0.441^2^
CPB time, minutes - median (IQR)	189 (121-253)	142.5 (103-190.5)	0.017^2^
Patient re-initiated on CPB - no. (%)	6 (15.8)	10 (13.2)	0.777^1^
Lowest intra-operative temperature, Celsius - median (IQR)	35.1 (33.9-35.9)	35.05 (34.6-35.8)	0.159^3^

**Figure 2:**
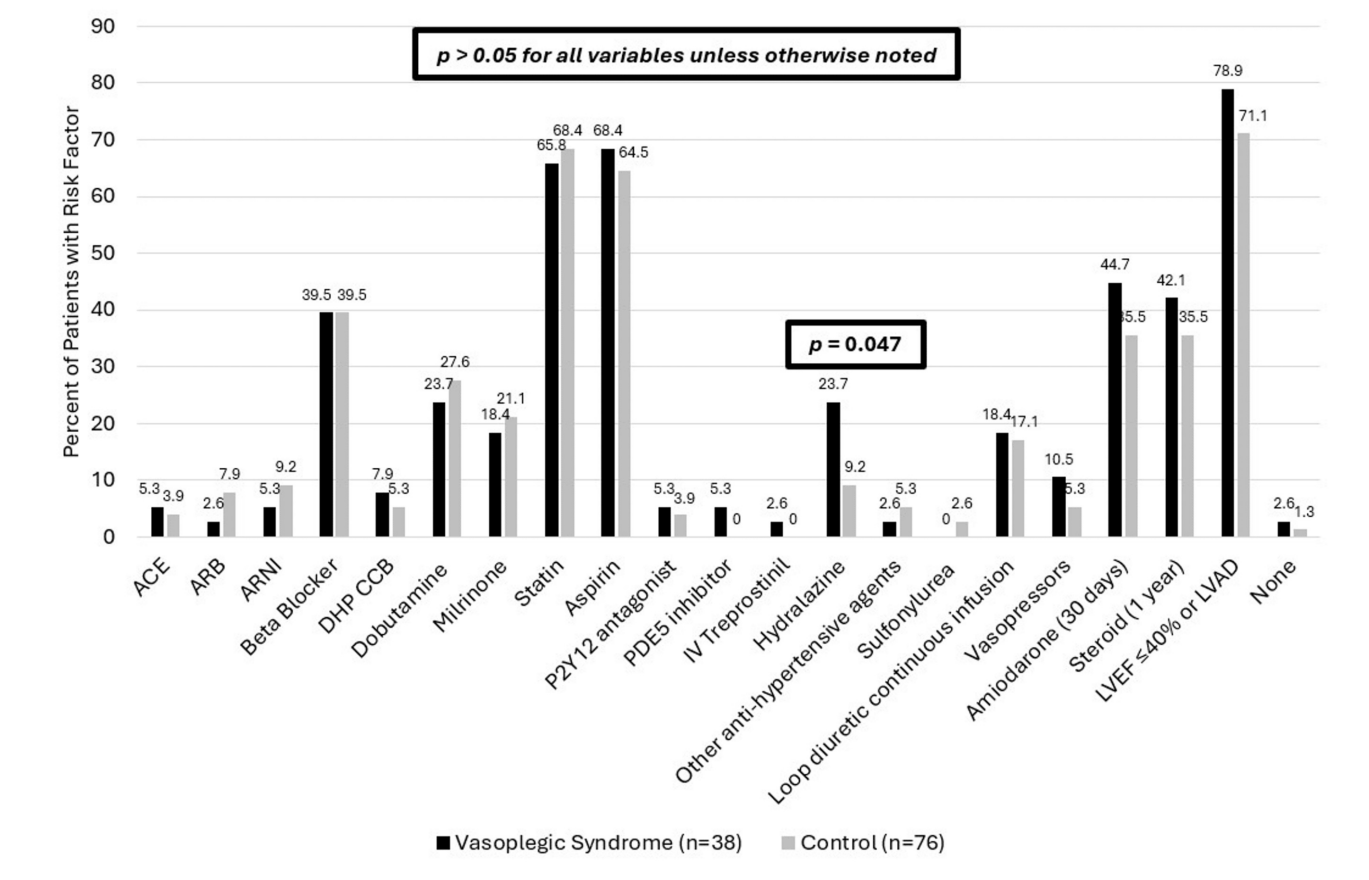
Incidence of preoperative factors assessed ACE, angiotensin-converting enzyme inhibitor; ARB, angiotensin II receptor blocker; ARNI, angiotensin receptor/neprilysin inhibitor; DHP CCB, dihydropyridine calcium channel blocker; IV, intravenous; LVAD, left ventricular assist device; LVEF, left ventricular ejection fraction; PDE, phosphodiesterase 5

The primary outcome was a correlation of risk factors of VS. When comparing the occurrence of these factors between groups, hydralazine was the only factor with a statistically higher occurrence in the VS group (23.7% and 9.2%, respectively, p=0.047). The rate of occurrence for the other factors can be seen in Table 2 and Figure 2.

**Table 2:** Comparison of preoperative risk factors ^1^Fisher exact p-value LVAD, left ventricular assist device; LVEF, left ventricular ejection fraction; VS, vasoplegic syndrome

Variable - no. (%)	VS (n=38)	Control (n=76)	P-value
Angiotensin-converting enzyme inhibitor	2 (5.3)	3 (3.9)	1.000^1^
Angiotensin II receptor blocker	1 (2.6)	6 (7.9)	0.421^1^
Angiotensin receptor/neprilysin inhibitor	2 (5.3)	7 (9.2)	0.715^1^
Beta blocker	15 (39.5)	30 (39.5)	1.000^1^
Dihydropyridine calcium channel blocker	3 (7.9)	4 (5.3)	0.684^1^
Dobutamine	9 (23.7)	21 (27.6)	0.822^1^
Milrinone	7 (18.4)	16 (21.1)	0.809^1^
HMG-CoA reductase inhibitors (statins)	25 (65.8)	52 (68.4)	0.833^1^
Aspirin	26 (68.4)	49 (64.5)	0.834^1^
P2Y12 antagonist	2 (5.3)	3 (3.9)	1.000^1^
Scheduled phosphodiesterase 5 inhibitor	2 (5.3)	0 (0.0)	0.109^1^
Intravenous treprostinil	1 (2.6)	0 (0.0)	0.333^1^
Hydralazine	9 (23.7)	7 (9.2)	0.047^1^
Other anti-hypertensive agents	1 (2.6)	4 (5.3)	0.663^1^
Sulfonylurea	0 (0.0)	2 (2.6)	0.552^1^
Loop diuretic continuous infusion	7 (18.4)	13 (17.1)	1.000^1^
Vasopressors	4 (10.5)	4 (5.3)	0.438^1^
Amiodarone (30 days)	17 (44.7)	27 (35.5)	0.415^1^
Steroid (1 year)	16 (42.1)	27 (35.5)	0.542^1^
LVEF ≤40% or LVAD	30 (78.9)	54 (71.1)	0.499^1^
None	1 (2.6)	1 (1.3)	1.000^1^

**Figure 3:**
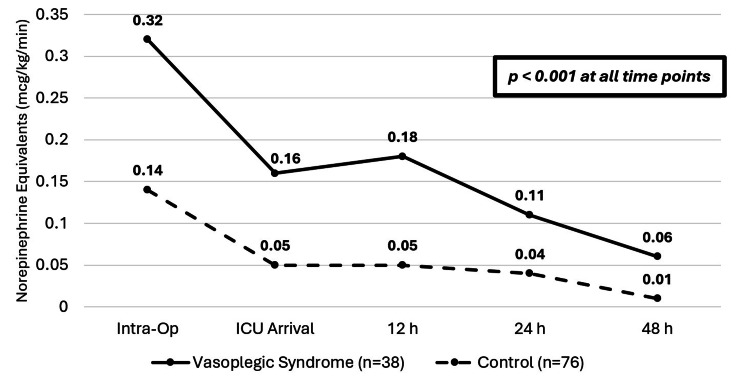
Median vasopressor requirements in NEE VS (n=38): solid line; control (n=76): dotted line VS, vasoplegic syndrome; NEE, norepinephrine equivalents

Secondary outcomes included vasopressor utilization, ICU length of stay, and hospital length of stay. Vasopressor utilization was higher at all time points in the VS group compared to the control group (Figure 3). ICU length of stay was longer in the VS group compared to the control group (seven days and four days, respectively, p=0.004), as was hospital length of stay (25 days and 23 days, respectively, p=0.028). Finally, survival to hospital discharge was higher in the control group, with only 28 (73.7%) patients in the VS group discharging to home or a step-down facility compared to 75 (98.7%) in the control group (p<0.001).

## Discussion

Development of VS is associated with high morbidity and mortality [3]. One known risk factor for the development of VS is prolonged CPB time [3,9]. This study showed a longer CPB time in patients with VS, which is consistent with the results of previous studies. Multiple studies sought to determine an association between specific medications and the development of VS, with conflicting evidence [3,9]. This study evaluated several of the previously evaluated medications, including ACE inhibitors, angiotensin II receptor blockers, angiotensin receptor/neprilysin inhibitors, beta blockers, and inotropes, and found no difference between groups. However, the low incidence of some of these factors could have contributed to a lack of difference. Use of hydralazine, both oral and intravenous administration combined, was statistically higher in the VS group. Hydralazine is an arteriole vasodilator, resulting in reduced peripheral resistance to decrease blood pressure and increase venous return, heart rate, and cardiac output [10]. Heart rate increases due to a compensatory release of norepinephrine and epinephrine mediated by baroreceptors. The proposed mechanism for hydralazine’s direct arterial vasodilatory properties includes myosin phosphorylation inhibition in arterial smooth muscle cells and inhibition of IP3-induced release of calcium in the sarcoplasmic reticulum. However, the precise mechanism of hydralazine has not been determined [10]. There is some evidence that hydralazine may enhance NO signaling, but this remains an area of ongoing research [11-13]. Hydralazine is rapidly absorbed, and peak concentrations are usually seen around one to two hours after oral administration [10]. It is highly protein-bound (88-90%), and serum concentration is dependent on acetylation, so responses can vary between patients. In patients with renal dysfunction, the half-life of hydralazine may be prolonged from the typically anticipated two to seven hours [10]. Although conclusions regarding the clinical significance of the finding of a higher incidence of hydralazine in the VS group cannot be drawn from this study, it is hypothesis-generating. This association could be due to the variable responses and duration of effect of hydralazine between patients or its potential mechanism of enhancing NO signaling.

This study showed higher vasopressor utilization in the VS group, which is to be expected as vasopressors are typically the first-line treatment. Those in the VS group also had longer ICU and hospital length of stays and poorer survival to hospital discharge, supporting the relationship between VS and high morbidity and mortality.

Limitations to this study include a retrospective chart review design and the potential for incomplete documentation. For example, charting of vasopressor dose adjustments may have been delayed, and mean arterial pressures at the time of treatment with methylene blue, hydroxocobalamin, or angiotensin II may not have been documented. The definition of VS utilized is not a standard definition, but was utilized to appropriately identify patients who experienced VS. Using the receipt of methylene blue, hydroxocobalamin, or angiotensin II within 48 hours of CPB initiation may limit the generalizability of our findings to those patients who experience VS but do not require the use of these agents. When matching patients who underwent CABG, the number of occlusions bypassed was not specified in the data collection. This study included fewer patients than initially predicted due to the 48-hour exclusion time post-CPB. Because of this, the sample size may not be enough to detect differences between groups and identify risk factors. Additionally, the initial intent was to perform a multivariate analysis to identify risk factors for developing VS. However, given the small margin of difference in variables between groups, such an analysis was not performed. Finally, the study timeframe included parts of the COVID-19 pandemic.

## Conclusions

In conclusion, VS is a disease state with high morbidity and mortality, especially for patients undergoing cardiac surgery with CPB. In this study, patients who developed VS had longer CPB times, which aligns with prior research identifying this as a risk factor for VS development. Hydralazine was the only pre-operative factor identified in this study with a higher incidence of usage in the VS group. This suggests a potential association between hydralazine use and the development of VS, which could be attributed to hydralazine's mechanism of enhancing NO signaling. The clinical significance of this difference cannot be drawn from the results of this study, but it supports further research into the interaction. The lack of differences observed with the other factors studied, including ACE inhibitors, beta blockers, and inotropes, may be due to insufficient statistical power and low incidences of these factors, highlighting the need for larger studies. These findings are hypothesis-generating, and future studies could further evaluate the relationship between hydralazine and the development of VS.

## References

[1] Lambden S, Creagh-Brown BC, Hunt J, Summers C, Forni LG (2018). Definitions and pathophysiology of vasoplegic shock. Crit Care.

[2] Fischer GW, Levin MA (2010). Vasoplegia during cardiac surgery: current concepts and management. Semin Thorac Cardiovasc Surg.

[3] Levin MA, Lin HM, Castillo JG, Adams DH, Reich DL, Fischer GW (2009). Early on-cardiopulmonary bypass hypotension and other factors associated with vasoplegic syndrome. Circulation.

[4] Datt V, Wadhhwa R, Sharma V, Virmani S, Minhas HS, Malik S (2021). Vasoplegic syndrome after cardiovascular surgery: a review of pathophysiology and outcome-oriented therapeutic management. J Card Surg.

[5] Blaha M, Blais M, Olson L (2023). The durability of intravenous hydroxocobalamin in vasoplegia. Cureus.

[6] Behlers QB, Buenger BK, Peitz GJ, Shostrom VK, Brannan SC (2025). Hydroxocobalamin dosing strategies for vasoplegic syndrome post-cardiac surgery: a retrospective cohort study. Cureus.

[7] van Vessem ME, Palmen M, Couperus LE (2017). Incidence and predictors of vasoplegia after heart failure surgery. Eur J Cardiothorac Surg.

[8] Khanna A, English SW, Wang XS (2017). Angiotensin II for the treatment of vasodilatory shock. N Engl J Med.

[9] Chawla LS, Busse L, Brasha-Mitchell E, Davison D, Honiq J, Alotaibi Z, Seneff MG (2014). Intravenous angiotensin II for the treatment of high-output shock (ATHOS trial): a pilot study. Crit Care.

[10] McComb MN, Chao JY, Ng TM (2016). Direct vasodilators and sympatholytic agents. J Cardiovasc Pharmacol Ther.

[11] Dulce RA, Yiginer O, Gonzalez DR, Goss G, Feng N, Zheng M, Hare JM (2013). Hydralazine and organic nitrates restore impaired excitation-contraction coupling by reducing calcium leak associated with nitroso-redox imbalance. J Biol Chem.

[12] Chirkov YY, De Sciscio M, Sverdlov AL, Leslie S, Sage PR, Horowitz JD (2010). Hydralazine does not ameliorate nitric oxide resistance in chronic heart failure. Cardiovasc Drugs Ther.

[13] Xu B, Bobek G, Makris A, Hennessy A (2017). Antihypertensive methyldopa, labetalol, hydralazine, and clonidine reversed tumour necrosis factor-α inhibited endothelial nitric oxide synthase expression in endothelial-trophoblast cellular networks. Clin Exp Pharmacol Physiol.

